# Mechanism of *Polygonum capitatum* intervention in pulmonary fibrosis based on network pharmacology and molecular docking technology: A review

**DOI:** 10.1097/MD.0000000000034912

**Published:** 2023-09-15

**Authors:** Zhiliang Fan, Xiang Pu, Lailai Li, Qian Li, Te Jiang, Liping Lu, Jingwen Tang, Mei Pan, Liyan Zhang, Yihui Chai

**Affiliations:** a School of Pharmacy, Guizhou University of Traditional Chinese Medicine, Guiyang, China; b School of Preclinical Medicine, Guizhou University of Traditional Chinese Medicine, Guiyang, China; c Technical Patent Department of Guizhou Weimen Pharmaceutical Co., Ltd., Guiyang, China.

**Keywords:** molecular docking, network pharmacology, pulmonary fibrosis, THL

## Abstract

Pulmonary fibrosis (PF) is a serious interstitial disease that includes diffuse collagen deposition of lung tissue. *Polygonum capitatum* Buch.-Ham. ex D. Don (THL) is a traditional vaccine that has antibacterial and anti-inflammatory effects. In this research, to investigate the mechanism of action of THL in the intervention of pulmonary fibrosis by network pharmacology and molecular docking related research methods, in order to provide a theoretical basis for expanding the scope of THL medication. A total of 49 active ingredients were analyzed and screened in *Cephalus cephalusis*, including 35 pulmonary fibrosis targets, and 10 key targets such as ALB, EGFR were screened after software analysis. The molecular docking results showed that there were 44 binding energies less than –3 kcal·mol^−1^ in the 60 docking results, indicating that most proteins had strong binding energies with compounds. The key targets of KEGG enrichment analysis were mainly enriched in 20 core action pathways, such as hemostasis-related pathway, regulation of kinase activity. This study shows that based on network pharmacology, the multicomponent–multitarget–multipathway effect of THL intervention in pulmonary fibrosis is discussed.

## 1. Introduction

Pulmonary fibrosis (PF) is a serious interstitial disease. Its pathological characteristics include diffuse collagen deposition of lung tissue, poor elasticity, weakening of the regeneration ability of pulmonary epithelial cells, and the destruction and fusion of alveolar structure, leading to airway remodeling, reduced lung ventilation, and ventilation. It seriously affects the quality of life of patients, and shortens the survival time of patients. Patients generally have a mortality rate of 50% for 3 to 4 years after diagnosis, and the treatment options are limited. Nidanib, pyrimidine, and acid inhibitors are recommended in the diagnosis and treatment guidelines for lung fibrosis, but the above drugs have adverse reactions and are expensive.^[1]^ At present, it is in the global pandemic of Corona Virus Disease 2019 (COVID-19). Epidemiological surveys show that severe patients are mostly accompanied by PF, so controlling the development process of PF is of great significance for treating the development of COVID-19.

*Polygonum capitatum* Buch.-Ham. ex D. Don (THL) is a perennial herb of THL and the main ingredient of Relinqing Granules (Guizhou Weimen Pharmaceutical Co., Ltd.). Relinqing granules have the effects of clearing heat and diasipation and diuresis. Relevant clinical reports show that Relinqing granules have a significant effect on urinary tract infections and other diseases. Some basic studies also show that Relinqing granules have the effects of reducing the expression of inflammatory factors, inhibiting bacterial growth, and antioxidant damage. Modern pharmacological studies have found that THL has a spectrum of anti-inflammatory effects, which is mainly dependent on Toll-like receptor 4, Phosphoinositide-3-kinase,mitogen-activated protein kinases, and other signaling pathways, and is related to inhibiting the expression of inflammatory factors and reducing the phosphorylation of related proteins It is worth noting that THL contains a variety of active ingredients such as flavonoids, lignin, and volatile oil, which have a certain anti-inflammatory effect, but at present, there is a lack of research on the pharmacological mechanism of THL interfering lung fibrosis.^[2]^

Network pharmacology research is a scientific theoretical method to analyze and predict the mechanism of drug-acting diseases by using network big data screening, bioinformatics analysis, and network visualization technology to analyze and predict drug-target-disease interaction networks.^[3,4,5]^ Since there is no mechanism and material basis for the treatment of lung fibrosis, this paper uses network pharmacological research methods to screen the chemically active substances, targets, and mechanisms of THL interfering with PF, to provide a scientific theoretical basis for the clinical application of the drug in the later stage.

## 2. Materials and methods

### 2.1. Screening of related ingredients and targets of THL

Through a large number of references, the chemical composition of THL is summarized, and the structure of each compound is drawn with ChemDraw software. Enter the structure of each compound drawn into the Swiss ADME database and specify that the criteria for selecting potential compounds are: GIabsortion is “High,” indicating that this ingredient has good oral bioavailability and is easy to absorb; the results of the 5 types of drug tests: Lipinski, Ghose, Veber, Egan, and Muegge have no less than 2 “yes.”

Subsequently, according to the results of the literature, if the pharmacological effect of the compound is highly correlated with PF, it is still retained and the final potential compound is obtained. Then use the PharmMapper database based on the drug-effect group matching method to obtain the core action targets of different potential compounds, select the target protein of NF (NormalizedFitScore) ≥ 0.9, and combine with relevant literature reports to synthesize the core action target of active compounds.

### 2.2. Disease target screening

To ensure the correctness and comprehensiveness of the target data, using “Pulmonary Fibrosis,” “lung fibrosis,” “Idiopathic pulmonary fibrosis,” “IPF” as the terms related to PF were the key search terms, Search GeneCards database, DRUG BANK database, OMIM database, and Therapeutic Target Database to obtain the core targets of PF diseases. Among them, the DRUG BANK database is a collection of clinical test drug data, GeneCards, OM, and Therapeutic Target Database based on literature data construction, Therefore, we use different databases for disease target point collection and obtain more comprehensive target point information. The collected target information is re-merged, and the potential targets of disease targets with pharmaceutical chemicals are uniformly standardized as Gene Symbol using the UniProt protein database and then map the two to obtain the target of potential intervention of THL in lung fibrosis.

### 2.3. THL composition-PF target

Upload the obtained intersection target to the STRING 11.5 database, select the Organisms as “Homo sapiens,” set the confidence to 0.40, conduct protein–protein interaction (PPI) network relationship analysis, and import the results into CytoScape3.7. 2 Software. The software is used to analyze the interaction relationship between proteins and obtain the potential functions of the relevant proteins.

### 2.4. THL composition and lung fibrosis target gene ontology functional enrichment and Kyoto Encyclopedia of Genes and Genomes pathway enrichment

The Metascape database is used for bioinformatics enrichment analysis of core targets, including gene ontology (GO) analysis of biological processes, molecular function and cell composition, and Kyoto Encyclopedia of Genes and Genomes (KEGG) pathways. The *P* value of the statistical analysis results is set to *P* ≤ .01, and the top 20 GO enrichment analysis results and KEGG pathways were selected according to the LogP value.

#### 2.4.1. Construction of component-target-channel network diagram.

Synthesize the results of bioinformatics enrichment analysis obtained by “1.4” and drug diseases obtained by “1.2” intersect the target and introduce them into Cytoscape 3.7.2 software to build: drug composition-target-channel network diagram. CytoScape3.7.2 software was used to obtain the topological analysis results of the potential chemical composition and targets of THL, including betweenness and degree, etc. According to the network topological analysis results, the core target and the main activities of intervention in lung fibrosis were obtained ingredients.

### 2.5. Molecular docking assessment

Selective analysis to obtain the medium value of THL composition-PF target network diagram ranked in the top 6. Download its pdb format through the RCSB database and convert the core compound to pdb format through Open Babel software. Proteins are dehydrated, liganded, hydrogenated, and stored in pdbqt format using pymol and Autodock Tools software. Treat and store potential compounds in pdbqt format by reducing energy and assigning ligand atomic types. Autodock software is used for molecular docking, and the binding energy Affinity is used as a reference for judging the binding activity.

### 2.6. Ethical approval

The current analysis does not require ethical approval, because our analysis only collects uploaded data information from the public database search.

## 3. Consequence

### 3.1. THL main active ingredient target prediction results

A total of 100 chemical structures related to THL were obtained from the relevant literature, and their main components were flavonoids, lignans and volatile oils, including terpenes, tannins and other components. Forty-nine active ingredients were screened by SwissADME, and the structure of the 10 most active ingredients is shown in Table 1. PharmMapper was used to predict and select targets with NF ≥ 0.9, for a total of 114 predicted targets after removing all repeat genes.

**Table 1:**
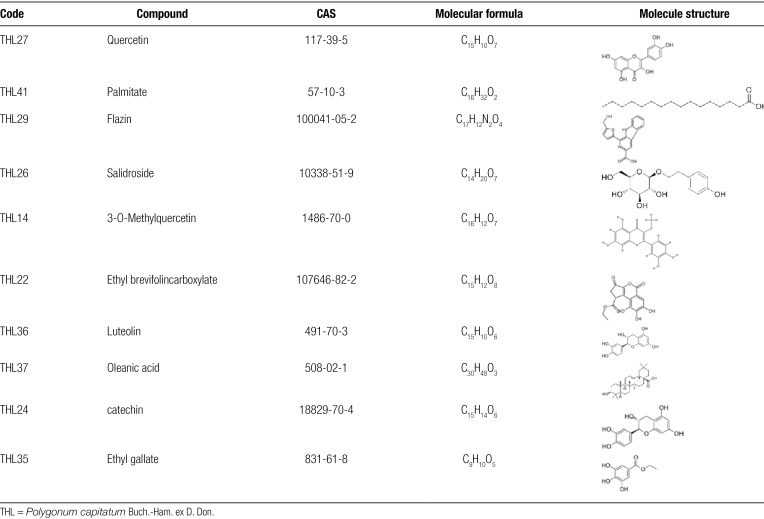
The 10 most active ingredients of Polygonum vulgaris.

### 3.2. THL intervenes in PF-related target acquisition

**Figure 1.**
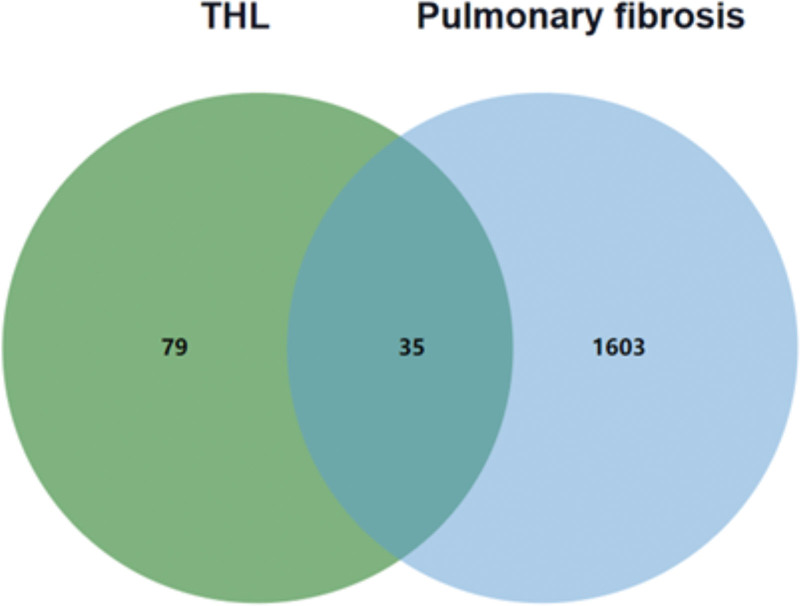
Venn diagram of the target of THL and the target of pulmonary fibrosis. THL = *Polygonum capitatum* Buch.-Ham. ex D. Don.

A total of 5807 targets related to PF diseases were obtained in the Genecards database, and the result of Score ≥ 4.50 was selected as the potential disease targets of PF, and 1449 related disease targets were collected. Combined with the Therapeutic Target Database, DRUGBANK, and OMIM databases, and deduplication and merger, 1638 PF-related disease targets were collected. 114 targets of THL active ingredient were intersected with 1638 PF-related targets to obtain 35 intersection targets, and the results were plotted as VENNA (Fig. 1).

### 3.3. PPI network construction

**Figure 2.**
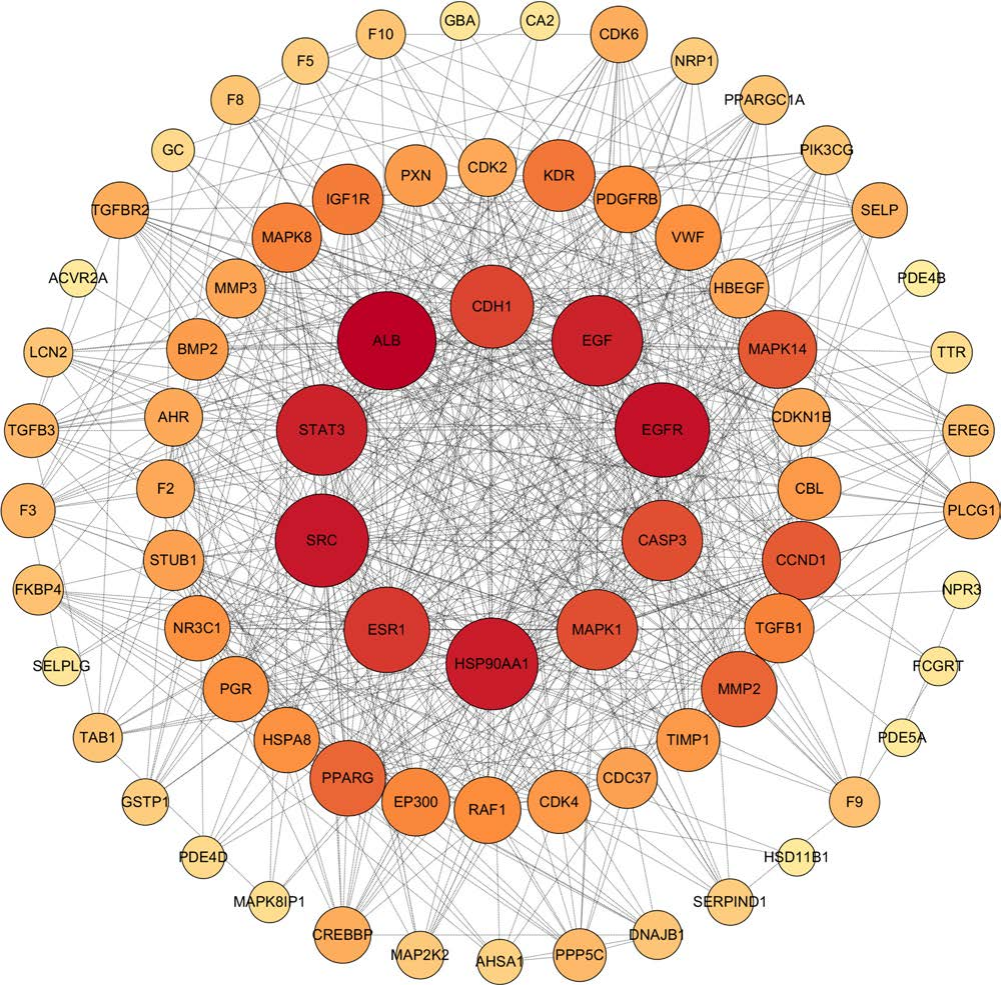
The PPI network of THL and pulmonary fibrosis targets. PPI = protein–protein interaction, THL = *Polygonum capitatum* Buch.-Ham. ex D. Don.

Upload 35 intersection targets to the String11.5 database for PPI analysis. The PPI network (Fig. 2) was constructed by using Cytoscape 3.7.2 software, and the network information was further analyzed to speculate that serum albumin (ALB), epidermal growth factor receptor (EGFR), etc. were the core targets.

### 3.4. GO functional enrichment and KEGG access enrichment

**Figure 3.**
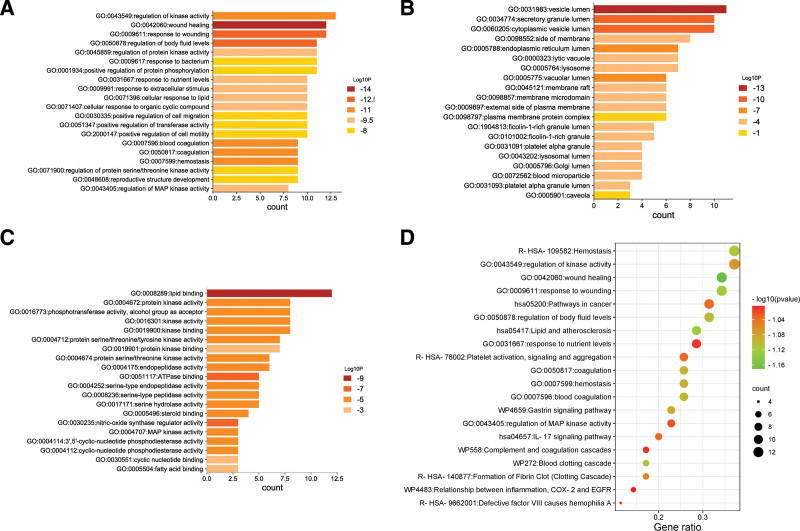
Enrichment analysis of potential targets of THL major components (A: GO-BP analysis; B: GO-CC analysis; C: GO-MF analysis; and D: KEGG analysis). BP = biological processes, CC = cell composition, GO = gene ontology, KEGG = Kyoto Encyclopedia of Genes and Genomes, MF = molecular function, THL = *Polygonum capitatum* Buch.-Ham. ex D. Don.

The Metascape database is used to analyze the bioinformatics enrichment of potential core targets (Fig. 3). Figure 3A shows that THL interferes in core biological processes related to PF: regulates lipid binding, regulates kinase activity, and regulates phosphotransferase Activity. The results of GO CC and GO MF are shown in Figures 3B and C. Figure 3D shows the signaling pathway of THL interfering with the top 20 PF in the results of KEGG pathway enrichment analysis.

### 3.5. Construction of THL component-target-pathway network

**Figure 4.**
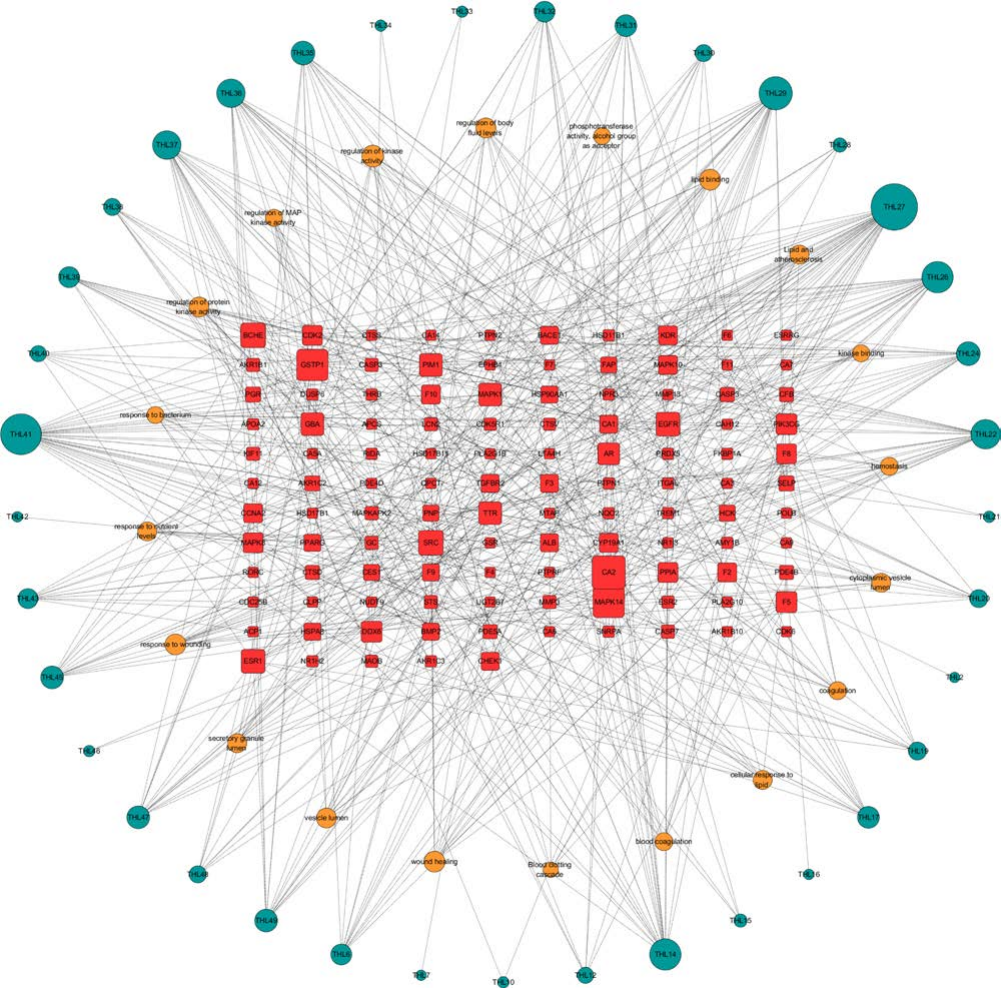
THL intervention pulmonary fibrosis component-target-pathway diagram. THL = *Polygonum capitatum* Buch.-Ham. ex D. Don.

The network diagram of “THL component-target-pathway” was constructed by using CytoScape3.7.2, and the topology information data of THL intervention in PF disease target network was analyzed, and the core chemical composition and target were further analyzed. As shown in Figure 4, this network consists of 172 nodes and 625 edges, the red nodes represent the potential targets of THL, the orange nodes represent the potential signaling pathways in THL, the green nodes represent the active ingredients of THL, the lines represent the relationship between different nodes, and the larger the node area and the darker the color indicate the greater its impact on PF.

Analysis of the network through Cytoscape found that each active ingredient of THL corresponds to multiple targets, and each target is connected to multiple components, indicating that the multiple components contained in THL may intervene in PF through multiple targets. Among them, THL27 (quercetin) degree value is 39, the intermediate degree is 0.139, and the tightness is 0.4 20, and THL27 is predicted to be the main component of THL intervention in PF, followed by THL41 (hexadecanoic acid) connection degree of 33, intermediate degree of 0.225, tightness of 0.433, THL29 (rosacealine) connection degree of 25, intermediate degree of 0.051, and tightness of 0.395 (Table 2). Carbonic anhydrase 2, mitogen-activated protein kinase 14, and Glutathione S-transferase P target genes ranked high in terms of network node degree, with connections of 25, 23and 23, respectively, predicting that kinase-binding was the core target of THL intervention in PF, and EGFR, estrogen receptor (ESR1), glucosylceramidase, transthyretin were also relatively important targets (Table 3). As can be seen from Table 4, different pathways are connected through common targets, indicating that THL may play a synergistic role in intervening PF through multiple pathways.

**Table 2:** Node characteristics of the main active ingredient network of THL.

Name	Degree	Betweenness centrality	Closeness centrality
THL27	39	0.13923015	0.42039801
THL41	33	0.22459592	0.43333333
THL29	25	0.05027364	0.39485981
THL26	23	0.08239552	0.37723214
THL14	23	0.03528678	0.38761468
THL22	21	0.04252777	0.38584475
THL36	20	0.03838579	0.36422414
THL37	20	0.04846092	0.37892377
THL24	15	0.02910837	0.34631148
THL35	15	0.01427912	0.36899563

THL = *Polygonum capitatum* Buch.-Ham. ex D. Don.

**Table 3:** THL main active ingredient target network node characteristics.

Name	Degree	Betweenness	Closeness centrality
CA2	25	0.15614927	0.29753521
MAPK14	23	0.07149997	0.40238095
GSTP1	23	0.07731984	0.35504202
SRC	16	0.01423756	0.37555556
BCHE	16	0.0389834	0.35208333
EGFR	15	0.02115095	0.38063063
ESR1	15	0.0230458	0.38235294
GBA	14	0.0284921	0.38940092
TTR	14	0.01905527	0.38235294
MAPK1	14	0.02161248	0.30505415

THL = *Polygonum capitatum* Buch.-Ham. ex D. Don.

**Table 4:** Pulmonary fibrosis target pathway enrichment results of THL intervention.

GO	Description	Count	Gene ID
R-HSA-109582	Hemostasis	13	ALB MAPK14 F2 F3 F5 F8 F9 F10 PIK3CG MAPK1 SELP SRC PDE5A
GO:0043549	Regulation of kinase activity	13	BMP2 CASP3 EGFR F2 GBA GSTP1 HSP90AA1 KDR PIK3CG PPARG SRC TGFBR2 PDE5A
GO:0042060	Wound healing	12	CASP3 MAPK14 F2 F3 F5 F8 F9 F10 KDR PIK3CG SRC TGFBR2
GO:0009611	Response to wounding	12	CASP3 MAPK14 F2 F3 F5 F8 F9 F10 KDR PIK3CG SRC TGFBR2
GO:0050878	Regulation of body fluid levels	11	MAPK14 F2 F3 F5 F8 F9 F10 GBA NPR3 PIK3CG SRC
GO:0009617	Response to bacterium	11	BMP2 CASP3 MAPK14 F2 GSTP1 LCN2 PDE4B MAPK1 MAPK8 SELP SRC
GO:0045859	Regulation of protein kinase activity	11	BMP2 CASP3 EGFR GBA GSTP1 HSP90AA1 PIK3CG PPARG SRC TGFBR2 PDE5A
GO:0001934	Positive regulation of protein phosphorylation	11	BMP2 EGFR F2 HSP90AA1 KDR PIK3CG PPARG MAPK1 SRC TGFBR2 PDE5A
hsa05200	Pathways in cancer	11	BMP2 CASP3 EGFR ESR1 F2 GSTP1 HSP90AA1 PPARG MAPK1 MAPK8 TGFBR2
hsa05417	Lipid and atherosclerosis	10	CASP3 MAPK14 HSPA8 HSP90AA1 MMP3 PPARG MAPK1 MAPK8 SELP SRC

THL = *Polygonum capitatum* Buch.-Ham. ex D. Don.

### 3.6. THL molecular docking screening

**Figure 5.**
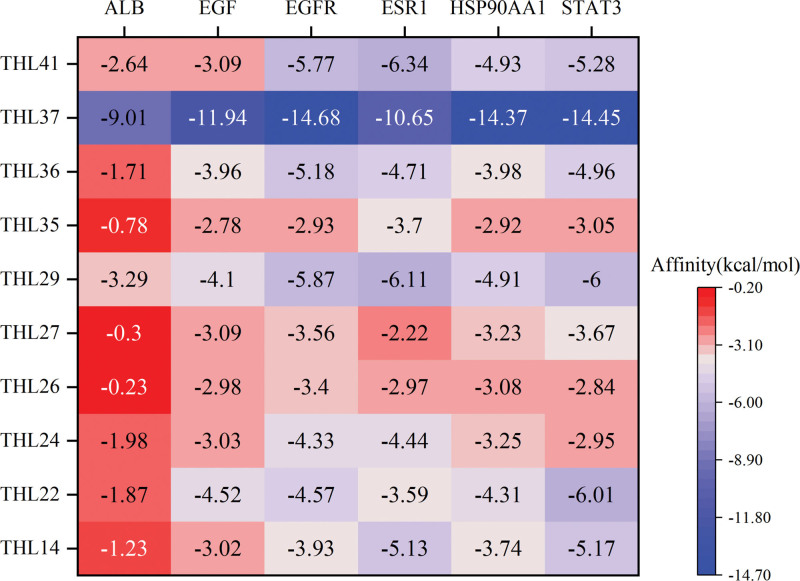
Molecular docking results.

**Figure 6.**
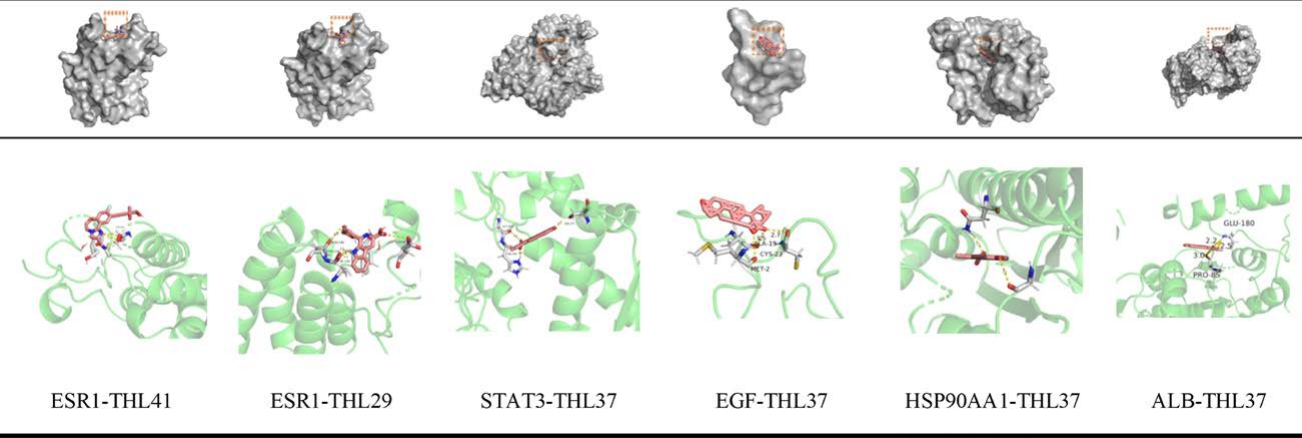
Molecular docking mode of some core compounds of THL. THL = *Polygonum capitatum* Buch.-Ham. ex D. Don.

The top 10 compounds in THL were molecularly docked with 6 core proteins ALB, EGF, EGFR, Heat shock protein HSP 90-alpha (HSP90AA1), ESR1, and Signal transducer and activator of transcription 3 (STAT3) obtained 60 groups of compound-protein docking results. Among them, there were 44 docking results of Affinity < -3 kcal·mol^−1^ and 5 of Affinity < −10 kcal·mol^−1^, among which EGFR-THL37 with the highest docking score was ^−^14.68 kcal·mol^−1^, indicating that the THL core compound had a strong binding activity with the core protein target. The docking results in this paper provide theoretical support for further THL-related experiments, and the docking results are shown in Figure 5 and the core docking mode is shown in Figure 6.

## 4. Discuss

### 4.1. Analysis of potential active ingredients

The main active ingredient of THL exerting anti-inflammatory and antibacterial effects is its flavonoid components, and the flavonoids in traditional Chinese medicine have a certain anti-fibrotic effect.^[6–8]^ Quercetin is one of the representative substances of THL flavonoids, and it has been found that quercetin can reduce the levels of malondialdehyde and hydroxyproline in mice with PF model, improve the total antioxidant capacity, regulate the expression of metalloproteinase-1 and then downregulate the expression of type III collagen, suggesting that quercetin has anti-fibrotic effects to a certain extent, and its mechanism may be related to the regulation of oxidative balance.^[9,10]^ Inflammation can participate in the occurrence and development of various stages of fibrosis, and excessive inflammation will promote the proliferation of a large amount of fibrous tissue and collagen deposition, thus becoming an important factor in the formation of fibrosis. Other studies have found that quercetin can achieve therapeutic effects on PF by regulating the release of various inflammatory mediators in the NF-κB signaling pathway.^[11]^ Transforming growth factor β1 (TGF-β1) is an important factor in promoting collagen deposition in lung tissue and plays an important role in the process of PF, and quercetin can inhibit the TGF-β pathway lung to alleviate fibrosis,^[12]^ in addition, quercetin can also affect the SphK1/S1P pathway and the Smad/β-catenin pathway,^[13]^ and these pathways have an important regulatory role in the course of PF.

In addition, the main active components of salidroside and luteolin in the intervention of PF were preliminarily screened out by network pharmacological research methods. Rhodiola rosea is one of the active substances of the traditional Chinese medicine Rhodiola rosea, and in recent years it has been reported to have antioxidant, anti-inflammatory, anti-aging, anti-cancer, and other effects.^[14]^ Rhodiolaside regulates oxidative stress responses in a variety of cells,^[15,16]^ which not only destroys lung tissue structure and affects alveolar ventilation, but also induces apoptosis and activates related signaling pathways to stimulate PF.^[17]^ JAK2/STAT3 and PI3K/AKT/MTOR signaling pathways are mainly involved in cell proliferation and differentiation and metabolic regulation and play an important regulatory role in the occurrence and development of PF.^[18,19]^ Salidroside can affect the expression of tyrosine-protein kinase JAK2 and STAT3 proteins, and regulate the PI3K/AKT/mTOR signaling pathway to achieve an intervention effect on fibrosis.^[20,21]^ NACHT, LRR, and PYD domains-containing protein 3 inflammasome can induce the release of a variety of downstream inflammatory factors, promote inflammation, promote oxidative stress, and promote the occurrence of fibrosis. Luteolin is widely found in a variety of traditional Chinese medicines, with anti-inflammatory, antioxidant stress response and other effects,^[22,23]^ luteolin can inhibit NACHT, LRR and PYD domains-containing protein 3 inflammasome activation, while reducing the level of interleukin-1 beta, Interleukin-18 thereby reducing the effect of PF.^[24]^ In addition, this study found that there are chemical components in THL that have not been reported to intervene in PF, such as cetanic acid, rosaceamine, etc., which can be further designed for experimental verification, and predict that this component may become a potential therapeutic direction for future research on THL intervention in PF.

The molecular docking results showed that the top 10 THL compounds all had a strong binding activity with the main protein targets, and the binding energy of THL37 (catechin) compounds to 6 proteins was <−9 kcal·mol^−1^, suggesting that this component may be the key component of THL in the treatment of PF.

### 4.2. Target analysis

The results of network topology analysis showed that there were 49 active components of THL, corresponding to 114 targets, including 35 common targets related to PF. Through the construction of the PPI network, it was found that ALB, EGFR, SRC, HSP90AA1, STAT3, ESR1, etc. have large degrees, which may be the most critical targets for the treatment of PF of THL. ALB is a binding protein synthesized by the liver and involved in various biological regulatory pathways such as inflammation and oxidative stress, which is often used as one of the markers for the evaluation of pneumonia, and when ALB is at a normal level, it is conducive to inhibiting the occurrence and development of fibrosis.^[25]^ The EGFR family is mainly involved in gene transcription and transduction, protein translation expression, apoptosis, and other processes, and it has been found that EGFR can transmit fibrotic signals downstream after activation, and has an important regulatory role in the physiological process of PF.^[26]^ SRC kinase can promote the activation of TGF, and at the same time it can regulate the proliferation of fibroblasts, induce pneumonia and PF, and related studies have shown that reducing SRC levels can reduce the proliferation and differentiation of myofibroblasts, control the apoptosis of epithelial cells, and play an important role in the formation of PF.^[27]^ HSP is a chaperone protein, which can bind to specific proteins to regulate cell proliferation, differentiation, and apoptosis, and studies have shown that HSP90 inhibitors have the function of inducing apoptosis in HSC cells, which may be by inhibiting TGF-β signaling pathways and oxidative stress response to play an intervention role in PF.^[28]^

### 4.3. Bioconcentration analysis

Through GO function enrichment analysis, it can be seen that the biological process of THL intervention in PF includes regulating lipid binding, regulating kinase activity, regulating phosphotransferase activity, etc., and through KEGG enrichment analysis, it can be seen that the signaling pathway of THL intervention in PF is mainly related to the regulation of nociception response and kinase activity, such as wound healing related signaling pathway, kinase activity regulation pathway, injury response related pathway, etc.

Hemostasis is an important pathophysiological element of PF, and in KEGG enrichment analysis, the Hemostasis signaling pathway is the signaling pathway with the most enriched genes, so it is speculated that it may be the key pathway for THL to intervene in PF. Patients with PF are often accompanied by pulmonary tissue bleeding, and endothelial and epithelial tissue damage will stimulate the activation of the coagulation response and promote the occurrence of PF when the patient’s wound heals.^[29]^ Kinase activity plays an important role in the treatment of PF. The extracellular regulated protein kinases (ERK) signaling pathway is mainly regulated by the expression of multiple protein kinases such as ERK1 and ERK2, and studies have shown that the high expression of ERK1 and ERK2 will promote the level of collagen synthase and thus promote the fibrotic deposition of extracellular matrix, resulting in the occurrence of fibrosis.^[30]^ The serine/threonine kinase (AKT) and [Pyruvate dehydrogenase (acetyl-transferring)] kinase isozyme are both protein kinases, and the combination of the two can induce AKT phosphorylation into the nucleus to regulate the expression level of related cell growth factors and promote the formation of fibrosis.^[31]^ Inhibition of ERK/AKT-related protein phosphorylation plays an important regulatory role in intervening in the occurrence and progression of PF.^[32]^ It was found that the abnormal expression of STAT kinase was closely related to lung injury and the formation of pulmonary inflammatory response, and its inhibition of inflammatory factors and Janus kinase (JAK) and STAT3 protein content was of great significance for improving the physiological state of lung interstitial, among which the JAK/STAT signaling pathway was mainly composed of tyrosine kinase JAK and STAT protein kinase, which combined to lead to PF.^[33,34]^

In summary, according to relevant experimental reports, the biological information pathways related to PF diseases are mainly related to nociception response and kinase regulation, suggesting that THL may intervene in PF by regulating JAK/STAT, Hemostasis, ERK, and other signaling pathways.

## 5. Summary

Modern pharmacological studies have found that Polygonum viviparum has a spectrum of anti-inflammatory effects, which is mainly dependent on TLR4, Phosphatidylinositol 4,5-bisphosphate 3-kinase, mitogen-activated protein kinase and other signaling pathways, and is related to inhibiting the expression of inflammatory factors and reducing the phosphorylation of related proteins.^[35,36]^ However, there are few studies on its treatment of PF disease. In this study, network pharmacology and molecular docking related techniques were used to preliminarily elucidate the mechanism of THL multi-component and multi-target joint regulation of PF through target prediction, protein interaction network, GO enrichment analysis, and KEGG pathway enrichment analysis. THL may act on biological targets such as ALB, EGFR, SRC, HSP90AA1, STAT3, EGF, and ESR1 through active ingredients such as quercetin, salidroside, luteolin, etc., and participate in the regulation of JAK/STAT, Hemostasis, ERK, and other signaling pathways to play a role in the treatment of PF. Based on scientific theories, this study speculates on the mechanism of THL intervention in PF, providing a theoretical reference for the discovery of new drug effects of THL. But there are still certain limitations in network pharmacology research, and the follow-up research team will continue to pay attention to the progress of THL pharmacological research. Mitochondria-targeted nano drug delivery systems are currently the focus of research, which can accurately deliver drugs and control drug release rate, and facilitate group modification and efficient loading of compounds.^[37]^ Our team will also conduct research based on green nanomaterials to target cells and mitochondria to explore the application value of targeted therapy of THL. In the future, our research team will continue to pay attention to the pharmacological research progress of THL and further explore the mechanism of THL in the intervention of PF combined with animal and cell experiments, to provide more effective treatment guidelines for the prevention and treatment of PF with traditional Chinese medicine and the diagnosis and treatment of COVID-19.

## Author contributions

**Conceptualization:** Xiang Pu, Te Jiang, Yihui Chai.

**Data curation:** Lailai Li.

**Formal analysis:** Xiang Pu.

**Funding acquisition:** Jingwen Tang, Xiang Pu.

**Investigation:** Liping Lu.

**Methodology:** Lailai Li.

**Resources:** Mei Pan.

**Software:** Te Jiang.

**Validation:** Liyan Zhang.

**Visualization:** Zhiliang Fan, Yihui Chai.

**Writing – original draft:** Zhiliang Fan.

**Writing – review & editing:** Zhiliang Fan, Qian Li, Yihui Chai.
